# Microstructure analysis and mechanical properties of phosphorus-reinforced ZCuPb20Sn5 alloy

**DOI:** 10.1038/s41598-019-49441-1

**Published:** 2019-09-16

**Authors:** Xiao-yan Ren, Xin Zhang, Xu Hong, Jiping REN

**Affiliations:** 1 School of Materials Science and Engineering, North University of China, Taiyuan, 030051 China; 2 Emergency Research Institute, Xinxing Cathay International, Beijing, 100070 China; 3 School of Materials Science and Engineering, Northern University of China, Taiyuan, 030051 China

**Keywords:** Metals and alloys, Design, synthesis and processing

## Abstract

An investigation was carried out to assess the effect of the P content on the microstructure and mechanical properties of ZCuPb20Sn5 alloy. Alloys of various compositions, (0.05, 0.1, 0.2, 0.3, 0.5% wt.% P) were melted in a melting furnace under 1200 °C and cast into metal mould, the hardness, strength and elongation of alloy castings which adding P or not in melting process were tested and the casting mircostructure was analyzed. The results show that the second phase appeared and gradually increased in amount with the content of P elements increased. Also, the microstructure of ZCuPb20Sn5 alloy was refined, and the average size of lead inclusions was reduced and formed a dispersed network of eutectoid inclusions.The addition of P had a beneficial effect on the microstructure and properties of ZCuPb20Sn5 alloy. The hardness and tensile strength of ZCuPb20Sn5 alloy increased, but the elongation increased at first, then decreased, when the P content increased. When the P content was less than 0.1 wt.%, the functions of phosphorous copper mainly was used as a deoxidizing initial gas, but when exceeded 0.1 wt.%, a second phase reinforcing particle formed with copper or nickel together, which improved the mechanical properties of the alloy. However, the elongation was lowered due to the brittle phosphide phase.

## Introduction

Cu alloys are widely utilized, due to the corresponding good casting performance, wear resistance, corrosion resistance and mechanical properties. Cu alloys are often used in the manufacturing of various machines to sustain heavy loads and high-speed operation of shaft sliding bearings, such as sleeves and bush bearings as well as in automobiles, ships, metallurgy, machinery and other industrial fields. With the rapid scientific and technological progress as well as economic development, the high requirements for Cu alloys increased rapidly^1^. High Pb-tin bronze alloys are currently widely used in the engine bearings with high-speed heavy load, excellent dry friction resistance, high thermal conductivity and fatigue resistance^2,3^. Currently, one of the main ways to improve the performance of tin-bronze alloys is to add alloying elements, such as rare earths, Ni, Pb, Fe, Mn, Al and P^4,5^. M. Aksoy^6^ and H. Turhan^7^ studied the matrix alloying method by adding Fe, Mn, Si, P, S and other elements to tin-bronze alloys, in which, the hard Mn_5_Si_3_ and Fe_3_S dispersed phases precipitated from the matrix, improved the sintering and abrasion resistance. Qi Zhan Jun^8^ studied the microstructure of high lead bronze alloy with different amounts of lead, S and rare earths, which demonstrated that the lack of S addition led to the severe lead segregation, occurring in the centrifugal casting of high lead bronze alloys. Also, the lead segregation was in the form of lumps and ribbons. The lead segregation was effectively controlled when S was added. With the addition of rare earths, the strip or block lead in the alloy changed into point-like or spherical lead. In this case, the point-like or spherical lead was uniformly distributed, while the degree of lead segregation decreased as the rare earth amount increased. C Nobel^9^ improved the tensile strength and wear resistance of lead-tin Bronze through the addition of traces of rare earths and Pb, which refined the microstructure and improved the mechanical properties. The tensile strength of the Pb-Sn bronze alloy reached 192 MPa when the pouring temperature, the contents of lanthanum-cerium-rare earth and P were 1250 °C, 0.2% and 0.5%, respectively. In the study of the effect of Fe traces on the microstructure and properties of semi-continuous casting of tin bronze, XY Mao^10^ demonstrated that the addition of Fe played an apparent role in grain refinement, reducing the columnar crystal sizes, along with the microstructure stress caused by the columnar crystals, while improving the subsequent processing performance.

P plays a specific role in high temperature alloys and it is generally regarded as a harmful non-metallic impurity^11,12^. However, few studies and reports on the role and mechanism of P in high temperature alloys have been reported. In recent years, significant attention has been paid to the role of P in alloys both domestically and abroad. AM Dubey^13^ and P Sahlot *et al*.^14^ studied the effect of P on the properties and segregation of superalloys. It was concluded that P, within a certain content range, could also significantly improve the life of alloys. Moreover, the addition of an appropriate amount of P could improve the creep and rupture properties of alloys^15^. But, the area of their application is limited because of low strength properties^16^. At present, the applications of P in Lead-Tin bronze alloys are seldom studied. Consequently, the reasonable amount control of P in the alloy has become a difficult problem. In the American standard^17^, the control of P content is related to the casting method. When sand casting, the P content does not exceed 0.5 *w*t.%, however continuous casting, the P content can be as high as 1.5 *w*t.%. In addition, for the United States, P is added to 0.1 *w*t.% in other national standards. For Japan, the P content in the residual elements is controlled at 0.1%, but he mentioned that in metal and centrifugal casting, the P content can be less than 0.5 *w*t.%.In this paper, the effects of P on the structure and properties of lead-tin bronze alloy were mainly analyzed on the basis of existing research results, in order to comprehensively understand the effect, function and mechanism of P on lead-tin bronze ZCuPb20Sn5, as well as to clarify the depth or process of the current research along with the existing problems, to design more targeted future research.

## Materials and Methods

The ZCuPb20Sn5 alloys with varying P content (0.05, 0.1, 0.2, 0.3 and 0.5 *w*t.%) were prepared by metal mould casting process. The bronze consists of 20% mass. of lead, 5% mass. of tin, 2% mass. of nickel, 1.75% mass. of zinc, and the rest is copper.

The raw materials chose electrolytic copper, lead ingot, tin ingot, zinc ingot, pure nickel (The purity is 99.99%) and P-copper alloy with a P content of 13.5 *w*t.%. High-melting nickel and copper initially placed into graphite crucibles and melted in a well-type melting furnace SG2-12-13. P was twice added as a deoxidizer. When the copper block was completely melted, 1/2-2/3 of P copper were added for initial deoxidization, whereas the remaining P-copper was added subsequently to all other alloying element additions. The alloying elements of Pb, Zn, Sn and P were added in sequence, according to the melting point values of the elements from high to low. When the melt temperature reached 1200 °C, quickly cast the liquid into a high temperature preheated metal mold and cooled to room temperature.

The specimens were cut, polished and macro-etched using the usual metallurgical technique. The hardness on the surface of sample was measured using a HB-3000B brinell hardness tester under the load of 250 kgf and duration of 30 s. The micro hardness was taken at different locations on the surface each specimen and an average value was calculated.Tensile tests were performed on a SANS-CMT5105 machine at ambient temperature and a tensile rate of about 2.4 mm/min. The microstructure was observed by a AXIO Scope.A1 and a SU5000 scanning electron microscope (SEM),Hitachi Inc. Tokyo, Japan, the average size of particles using the photograph of the microstructure.The compositions of every phase were determined by a SHIMAJZU energy dispersive spectrometer (EDS).The phase was analyzed via X-ray diffraction (XRD) using CuKα radiation, and the diffraction angle (2θ) varied from 20° to 80°. The crystalline phases were identified with JCPDS database cards.The X-ray diffraction with the applied voltage of 45 kV and current of 40 mA was also used to measure the pole fifigures.

## Results and Discussion

### Microstructure

**Figure 1:**
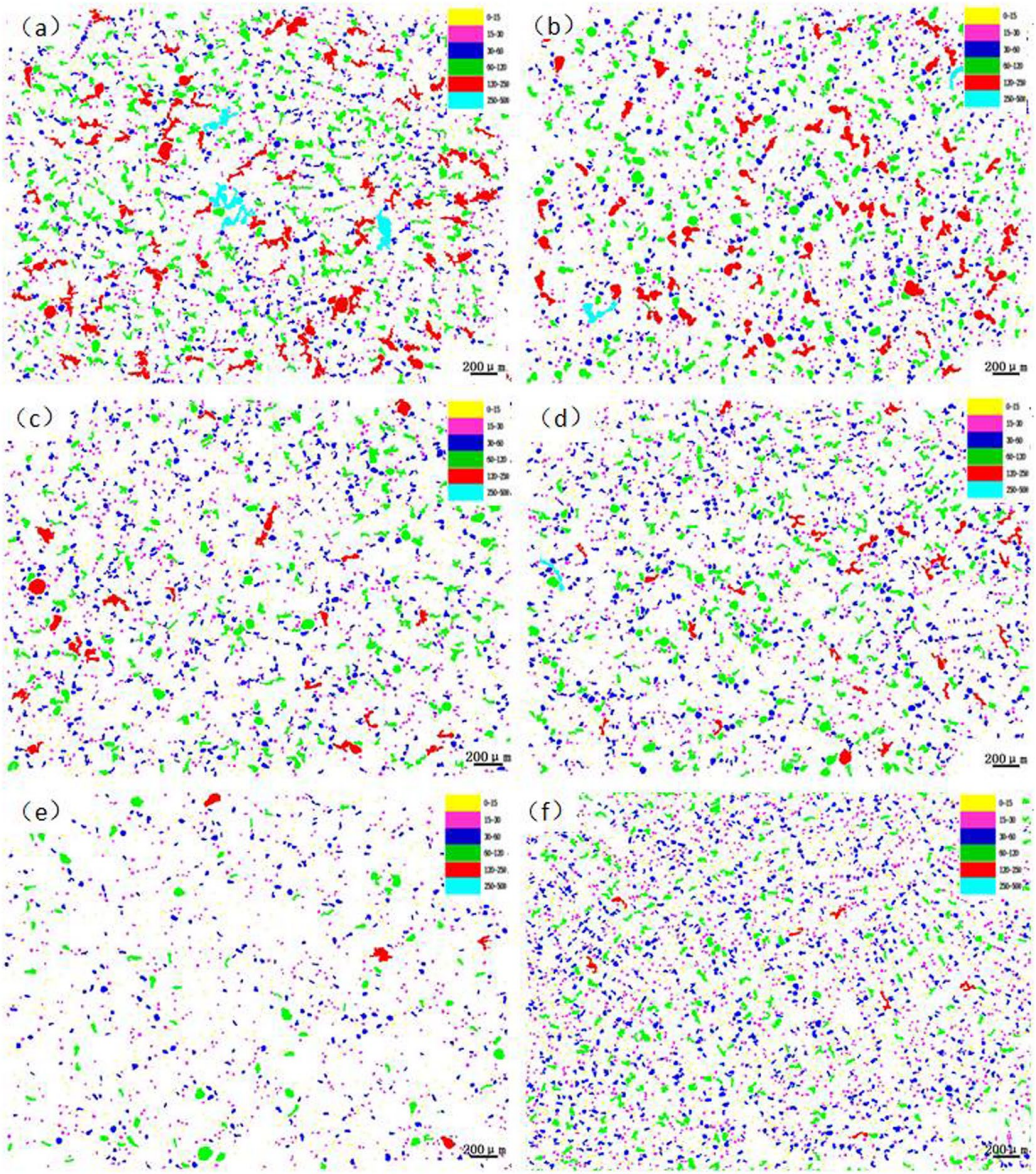
Changes of lead particle size distribution in lead tin bronze with different P contents: (**a**) P = 0.0 *w*t.%; (**b**) P = 0.05 *w*t.%; (**c**) P = 0.1 *w*t.%; (**d**) P = 0.2 *w*t.%; (**e**) P = 0.3 *w*t.%; (**f**) P = 0.5 *w*t.%.

**Figure 2:**
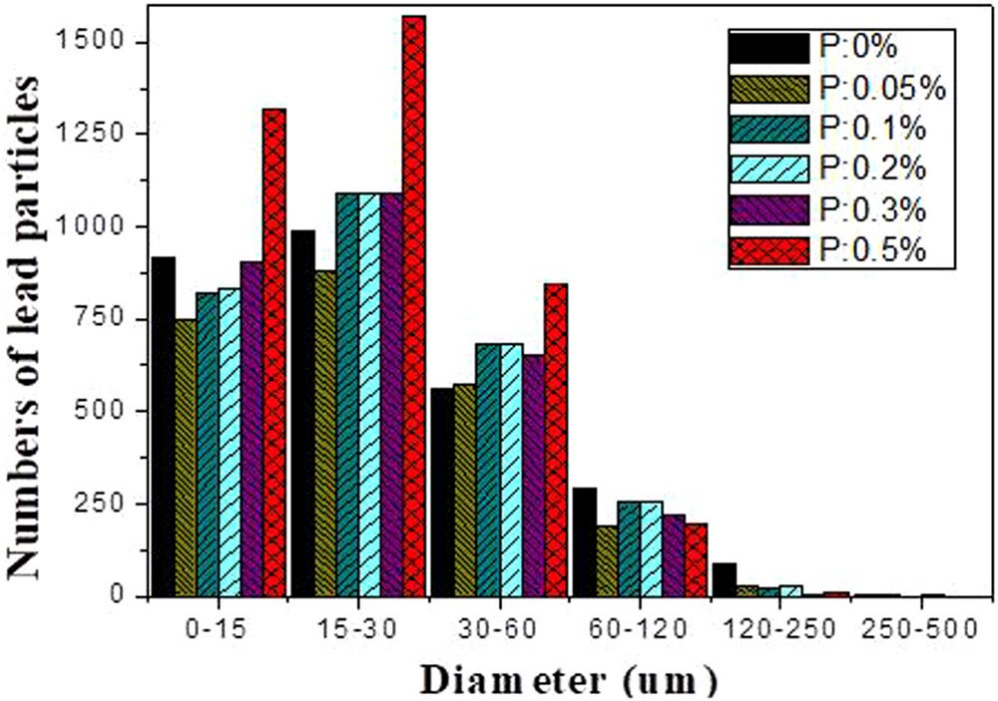
Lead particle number distribution map.

After the sample was polished, it was observed under a microscope. And the lead particles were evaluated by the graphite particle size evaluation standard. The picture of the lead particles and the data was extracted as shown in Figs 1 and 2. The metallographic phase under the field of view of 50 times was selected for evaluation and analysis. The particles of different colors in the Fig. 1. represented lead particles, which are distinguished by different colors according to different sizes. It could be observed that the large lead particles gradually disappeared and transformed into very small particles as the P content increased.

When no phosphorus was added, the number of particles in the range of 250–500 um is 4, the number reduced to 2 when 0.05 wt.% of p content. When 0.1 wt.% of P added, there was no precipitation of large particles of lead. Although there was a lead particle with the content of 0.2 wt.%, the morphology was small. It is observed that after the addition of phosphorus, not only the large particles were reduced, but also the morphology of the large particles was much smaller than when the P was not added. When the content of P added was more than 0.2 wt.%, the lead particles of the range of 250–500 um were not present; There were 84 lead particles in the range of 120–250 um in diameter no P, but the number reduced to 29 when P was added 0.05 wt.%. Then, When the P increased, the particles gradually decreased. There were only 7 lead particles in this range, when the amount of P added was 0.5 wt.%. As could be seen from the Fig. 2, the amount of lead particles gradually increased with the increased of phosphorus, especially when it increased to 0.5 wt.% in the range of 0–15 um and 15–30 um. The number of lead particles were the most, and the form was also the smallest. From this data, it could be concluded that the addition of phosphorus could refine the morphology of lead particles,convert large irregular lead particles into small spherical particles, and the spheroidization effect was better.

**Figure 3:**
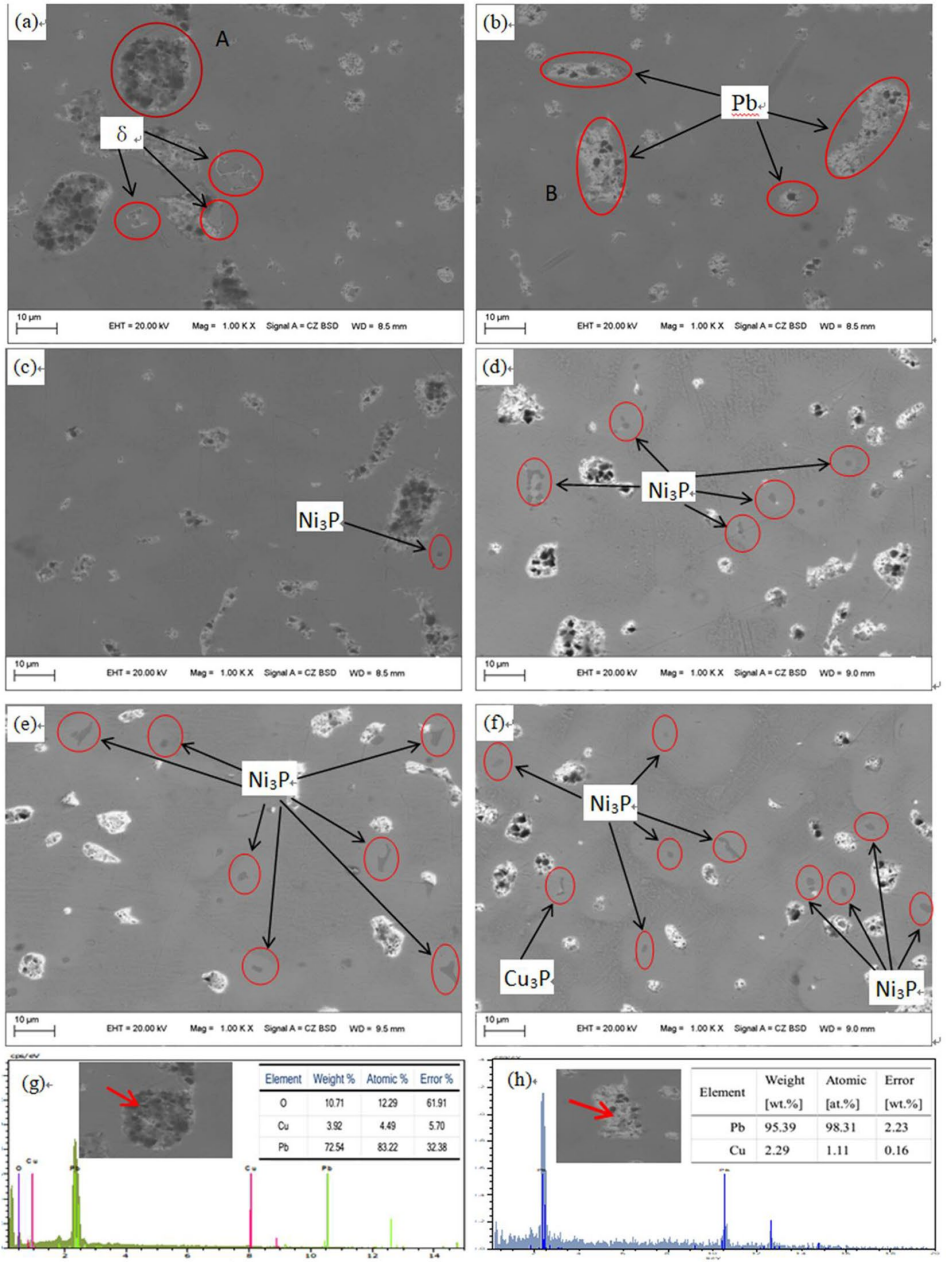
Microstructure of ZCuPb20Sn5 with different content of P: P = 0.0 wt.%; (**b**) P = 0.05 wt.%; (**c**) P = 0.1 wt.%; (**d**) P = 0.2 wt.%; (**e**) P = 0.3 wt.%; (**f**) P = 0.5 wt.%; (**g**) EDS of A; (**h**) EDS of B.

The SEM microstructures of the ZCuPb20Sn5 alloy with different P contents (0, 0.05, 0.1, 0.2, 0.3, 0.5 wt.%) are presented in Fig. 3(a–f), respectively. The gray matrices were the α solid solution with copper as the matrix, while the white-gray dendritic massive structure was a tin-rich solid solution, the (α + δ) phase with copper and tin as the matrix. The fine white particles were the lead particles. The large white massive particles were the grown lead particles, which were embedded in microscopic pores.

It could be observed from Fig. 3(a), without the addition of P, a high amount of lead was gathered at the microscopic pores and formed large particles before it could be dispersed, which led to the uneven distribution and high segregation of lead. In Fig. 3(b), the large lead particles decreased in size with 0.05 wt.% of P added, the large spherical particles started to be decomposed into small worm-like particles. The white-gray massive (α + δ) eutectoids were distributed irregularly and inhomogeneously.When the P content was 0.1 wt.% as shown in Fig. 3(c), the large lead particles apparently decreased in size and continued to transform large spherical into worm-like. One portion of the white-gray bulk (α + δ) eutectoid was irregularly and unevenly distributed, whereas the other part was evenly distributed among dendrites.When the P was 0.2 wt.% in Fig. 3(d), the spherical particles had basically transformed into vermicular particles. The volume of white-gray bulk (α + δ) eutectoids increased and distributed among the dendrites. Also, the dendrites became coarse.When the P was added to 0.3 wt.%, the Pb particles changed from wormlike to relatively small spherical particles and the gray massive (α + δ) eutectoid was distributed in the tissues producing chrysanthemum-like uniformity and irregularity. Moreover, the segregation of Pb particles was not apparent.

As the P content increased, the large lead particles became wormlike and consequently decomposed into spherical particles, evenly distributed in the tissue, reducing the segregation of lead. When the P-Cu alloy was not added, certain bulk δ matrix compounds could be faintly observed, and the large bulk lead particles in amount could be observed. When the P content was 0.05 wt.%, the Ni_3_P phase was not observed, the large lead particles diminished and the microstructure was relatively fine and uniform. When the content of P was 0.1%, the Ni_3_P phase with fine black-gray vermicular strips was barely visible.When the P content was 0.2 wt.%, the Ni_3_P phase increased in size and number, and the lead particles were small spheres without large aggregates. As the P content increased to 0.3 wt.%, the Ni_3_P phase with fine black-gray vermicular strips was very numerous and the shape had changed. When the P content is 0.5 wt.%, the second phase is seen more, and Cu_3_P phase appeared with Identical morphology.

From the aforementioned microstructure, it could be concluded that the addition of P reduced the segregation of lead, leading to a homogeneous matrix microstructure. However, when the content of P exceeded 0.1 wt.%, the volume of gray bulk (α + δ) eutectoid gradually increased, and the dendrites gradually became coarse. The solubility of P in solid solution was 0.1 wt.%, the eutectic structure (α + Ni_3_P) could be formed when the content of P exceeded 0.1 wt.%. The Ni_3_P phase was hard and brittle, often composed of binary and ternary eutectic phases with α and δ phases.

EDS spectra analysis of ZCuPb20Sn5 alloy prior to and following modification with P are presented in Fig. 3, respectively. Figure 3(g) is the EDS scan data at A in Fig. 3(a), and Fig. 3(h) is the B in Fig. 3(a). Comparing the two figures, it can be seen that large lead particles appeared in the tissue without P addition, and there was oxygen inside. However, after adding 0.05 wt.% of P, the large lead particles disappeared. And the relatively large lead particles contained only lead but no oxygen. It means that under the condition of deoxidizing and degassing with P, the lead particles adsorb a large amount of oxygen during the solidification process, and thus grow up, resulting in coarse and uneven tissue. The gas in the liquid can be completely removed with a P content of 0.05 wt.%, so that the solidification of lead in the alloy solution can be carried out in an orderly manner. As the P content continues to increase, a distinct second phase Ni_3_P appears in the structure, as shown in Fig. 3(c–f) and Fig. 3 in the red circle. It can be seen that when the amount of P added is less than 0.1 wt.%, the Ni_3_P phase is not observed in the structure, and when the amount is more than 0.1%, Ni_3_P gradually increases, and grows into a small wormlike shape. Moreover, when no P-Cu is added, a plurality of white-gray block-like δ phases appear in the structure, which is the solid solution based on the electronic compound of Cu_31_Sn_8_. But after the addition of P-Cu, the δ phase is reduced or even eliminated.

It is indicated that phosphorus in ZCuPb20Sn5 alloy liquid not only plays the role of deoxidation and degassing, but also can generate second phase reinforcing particles. When the P content is less than 0.1 wt.%, it mainly plays the role of deoxidation and degassing. When the p content is greater than 0.1 wt.%, in addition to deoxidation and degassing, the form of lead particles can be refined. The average size of lead inclusions was reduced and formed a dispersed network of eutectoid inclusions.

### Mechanical properties

**Figure 4:**
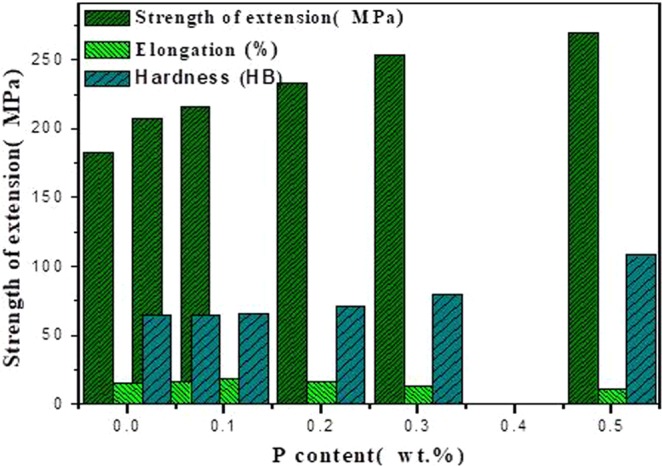
Mechanical properties curve.

The elongation and tensile strength of ZCuPb20Sn5 alloy with different P contents were measured through experimentation, as presented in Fig. 4. As the P content increased, the tensile strength and hardness of ZCuPb20Sn5 alloy presented an increasing trend. The tensile strength of ZCuPb20Sn5 alloy was 183.19 MPa without the phosphor copper alloy addition. When 0.3 wt.% of P was added, the tensile strength reached 253.48 MPa, which was increased by 70.29 MPa compared to the alloy without P-copper. The Brinell hardness of ZCuPb20Sn5 alloy with different P contents was measured through experimentation. Each hardness value was measured five times and the average value was obtained. when the P content was 0.05 wt.%, the hardness of ZCuPb20Sn5 alloy was basically unchanged from 64.2HB to 64.5HB. When the P content was 0.3 wt.%, the hardness of ZCuPb20Sn5 alloy increased to 79.58HB.The strength and hardness of ZCuPb20Sn5 alloy casting prepared by adding P 0.5 wt.% in melting process are 269.46 MPa and 108.18HB respectively, comparison with the strength and elongation of non-P refiner are 183.19 MPa and 64.2HB separately, they were increased by 47.1% and 68.5% respectively. The elongation of ZCuPb20Sn5 alloy increased first and consequently decreased as the P content increased. When the P content was 0.1 wt.%, the elongation of ZCuPb20Sn5 alloy reached the maximum of 18.65%. Following, the ZCuPb20Sn5 amount gradually decreased as the P content increased.When the P content was 0.3%, the elongation of ZCuPb20Sn5 alloy decreased to 12.78%. When the P-Cu alloy with a mass fraction of 0.5%.wt was added, the elongation of ZCuPb20Sn5 alloy decreased to 11.65%.

It was known that the addition of P improved the ZCuPb20Sn5 alloy hardness^18^. The Ni_3_P and Cu_3_P compounds were formed through the P-Cu alloy addition to the melt. The compounds were hard and evenly distributed at the grain boundaries, which inhibited the segregation of lead. In this way, on the one hand, Ni_3_P compound inhibited the movement of dislocations, while as the number of Ni_3_P compound particles increased, the resistance to dislocation motion increased, resulting in the alloy hardness increase. On the other hand, the Ni_3_P and Cu_3_P particles were uniformly distributed at the grain boundaries, resulting in the formation of several crystalline cores during solidification, which could refine the grain and produce fine grain strengthening. For a certain range, as the P content increased, the effect of fine grain strengthening became more apparent, consequently enhancing the ability of the matrix to resist plastic deformation. This led to the increase of hardness and wear resistance of lead-tin bronze alloy. Therefore, as the P content increased, the hardness increased at the macro level.

The solidification segregation of deformed superalloys was basically eliminated or highly reduced subsequently to hot working and heat treatment. Simultaneously, the P atoms were segregated at the grain boundary, changing the bonding relationship among main elements on the grain boundary, forming certain large clusters. In addition, the bonding force among atoms increased, improving the grain boundary strength, while changing the precipitate morphology at the grain boundary^15^. It could be preliminarily determined that as the P content increased, a high amount of Ni_3_P and a little Cu_3_P phases occurred, and owing to the presence of several phases, it can contribute to realisation of not only grain boundary strengthening due to the grain refinement, but also dispersion hardening by Orowan mechanism^19^, which consequently improving the tensile strength of ZCuPb20Sn5 alloy. When the content of Ni_3_P and Cu_3_P continued to increase, the thermal brittleness increased and the elongation decreased.

### Analysis

Lead is not dissolved in copper, and its solid solubility is also small in copper alloys. Lead and copper can form a fusible eutectic structure. The lead below 38% is immiscible with copper in liquid state, and can form a metamorphic structure when solidified. In the solid state, lead is distributed in the elemental state in copper and can be distributed in the crystal and the grain boundary. In lead-containing copper alloys, lead at the grain boundaries can be transferred into the crystal during phase change or recrystallization. The finer the particle of lead, and the more uniform the distribution, the better the performance.

P is a good deoxidizer for copper alloys, which increases the fluidity of the alloy and improves the process and mechanical properties of tin bronze. However, excessive addition will increase the degree of reverse segregation. The limit solubility of phosphorus in tin bronze is 0.15 *w*t.%. When too much, a + δ + Cu_3_P ternary eutectic will be formed. The binary phase diagram of copper and phosphorus^20^ shows that there is a eutectic reaction at 714 °C, that is L8.4% → α1.75% + Cu_3_P, and the solid solubility of phosphorus in copper decreases rapidly with the decrease of temperature, 0.6% at 300 °C and 0.4% at 200 °C.

**Figure 5:**
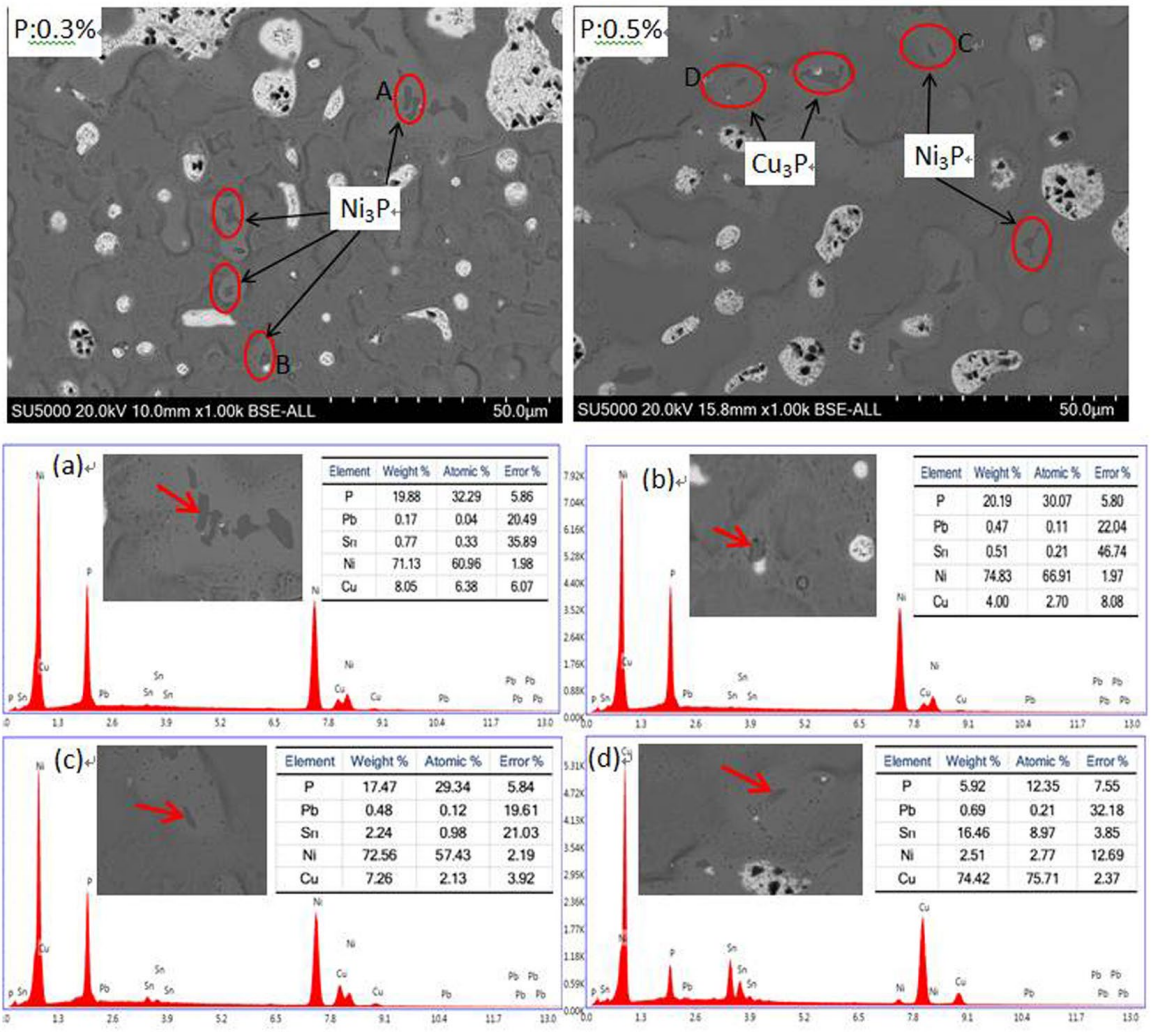
SEM images and EDS of ZCuPb20Sn5 with different content of P: (**a**) EDS of A; (**b**) EDS of B; (**c**) EDS of C; (**d**) EDS of D.

In order to prove the phosphide phase more clearly, the SEM micrographs of casted ZCuPb20Sn5-xP (x = 0.3 and 0.5) alloys were researched. Exemplary and the Ni_3_P and Cu_3_P phase were shown in Fig. 5. Combined with the energy spectrum, it could be observed that the gray massive microstructure A, B and C were mainly the Ni_3_P phase. Similarly, the dark gray mass D was mainly the Cu_3_P phase,which was similar characteristics in terms of Ni_3_P phase size and morphology. It should be noted that, on this high P amount level, the ultra-fine phosphide phase almost occurs as eutectoid and embeds in the rich tin (α + δ) eutectoids. When the amount of phosphorus added is 0.3%, the phosphide phase is mainly Ni_3_P. When the phosphorus content is increased to 0.5%, part of the phosphorus reacts with nickel dissolved in copper to form Ni_3_P, and the remaining reacts with copper to form Cu_3_P. Therefore, the phosphide phase of the ZCuPb20Sn5–0.5 P alloy is mainly Ni_3_P and Cu_3_P phases.

The ZCuPb20Sn5 alloy consists mainly of α solid solution, Pb and the (α+δ) eutectoids and it was found that the major secondary phase in the alloy was Ni_3_P. The α phase is the solid solubility of tin in copper, has a face-centered cubic lattice, has good plasticity due to tin. Dissolved in copper to produce solid solution strengthening, thus having a certain strength.

**Figure 6:**
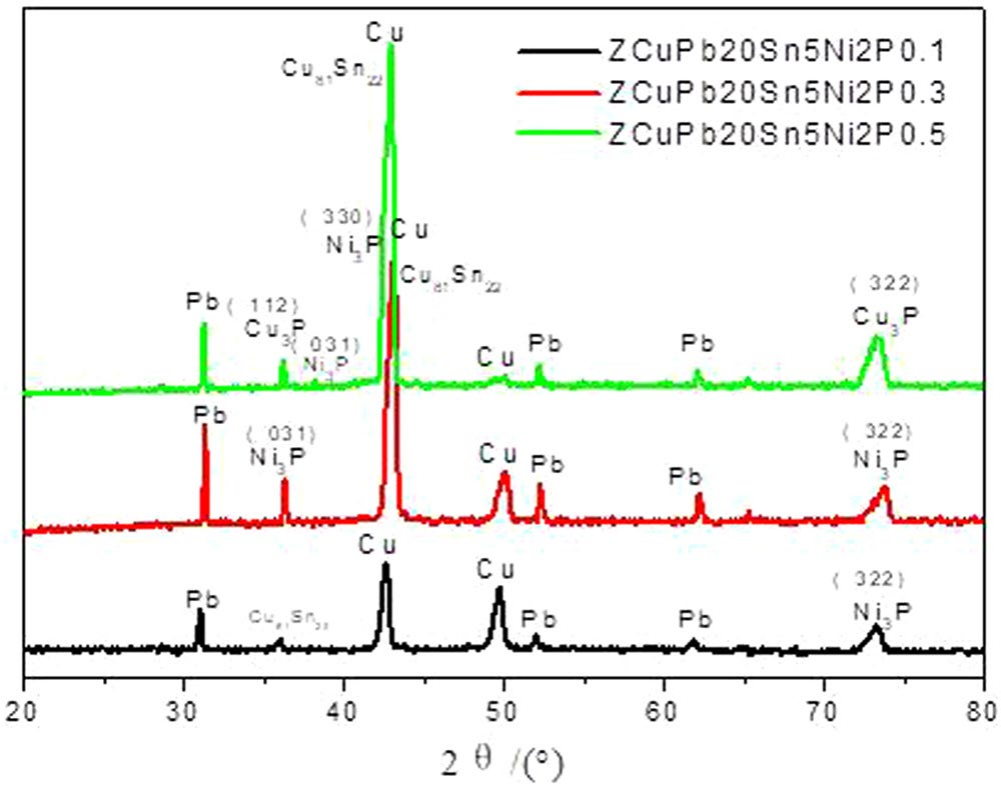
XRD analysis of ZCuPb20Sn5 alloy with different P.

**Figure 7:**
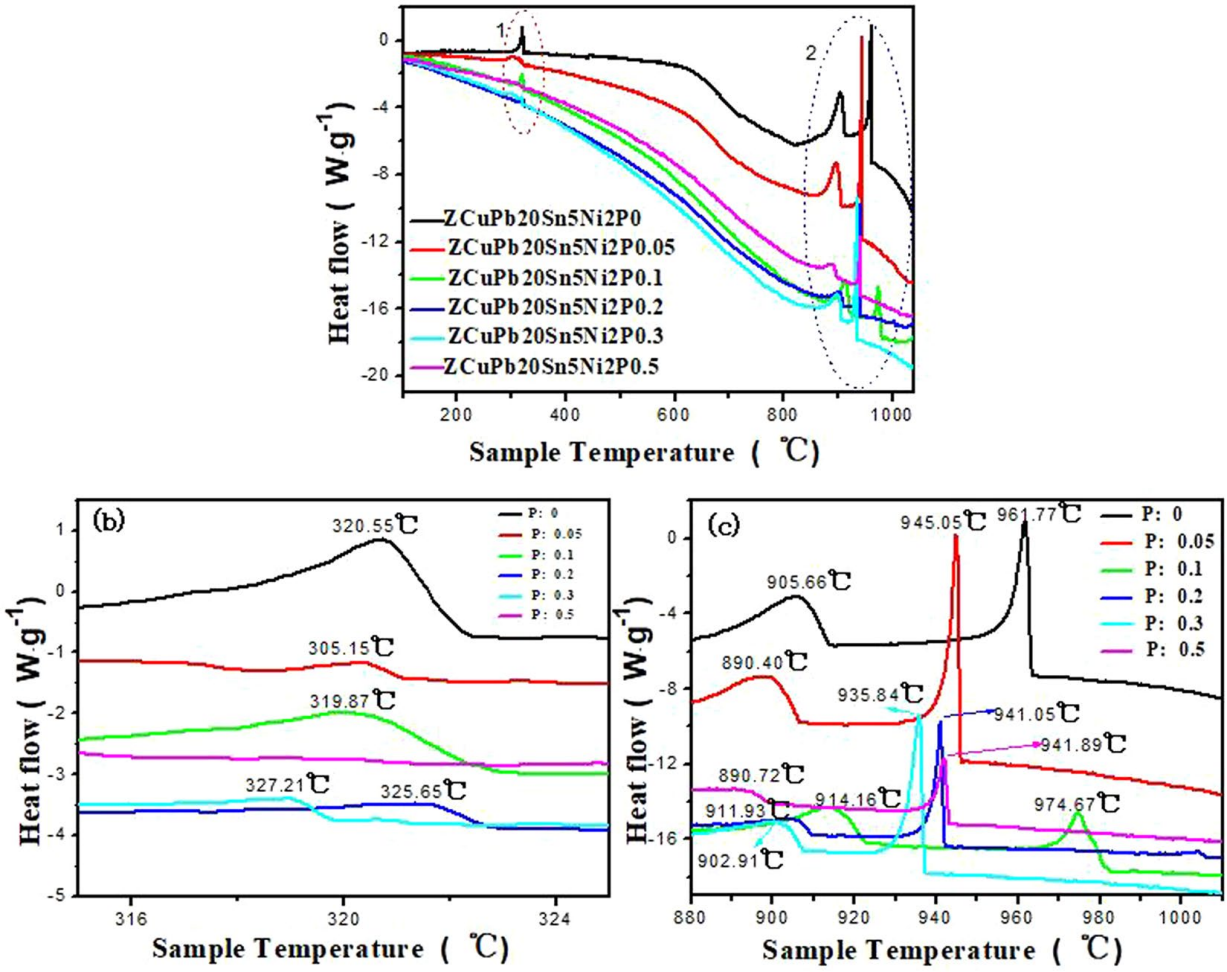
DTA analysis of ZCuPb20Sn5 alloy with different P; (**b**) DTA of 1; (**c**) DTA of 2.

To determine the effects of P on the alloy phase type and chemical composition of the second phases, the XRD and DTA spectrum analysis of the samples were carried out (Figs 6 and 7).

All the XRD patterns exhibit the major one series of peaks corresponding to a face-centered-cubic (FCC) phase (α-phase) attributed to Cu solid solution. That is in virtue of the quick cooling of samples in air. The lead phase appears mainly at four peaks, mainly at 31.305° (111) plane, 36.266° (200) plane, 52.228° (220) plane, and 62.119° (311) plane. Parameter of the α-(Cu) phase is evaluated from the positions of (111) diffraction peaks. The δ phase appears mainly on the 73.195° azimuth (022) plane.With the increase of P content, the intensity of diffraction peak increases.

The second phase is mainly the Ni_3_P phase. By varying different amounts of P, the Ni_3_P phase fraction increases as well as its diffraction peaks (BCT phase) emerge. When the p addition amount is 0.1%wt.%, a little Ni_3_P phase appeared on the 35° azimuth (031) plane, and the peak is very low. The amount of P added is 0.3 wt.%, the phosphide phase is mainly Ni_3_P. Ni_3_P phase appears on the 36.418° azimuth (031) plane, and the peak is high, and the main second phase is Ni_3_P, no Cu_3_P phase for the ZCuPb20Sn5-0.3P alloy. When the P content is increased to 0.5%wt.%, part of the P reacted with nickel dissolved in copper to form Ni_3_P, and the remaining formed Cu_3_P with copper. Therefore, the phosphide phase of the ZCuPb20Sn5-0.5 P alloy is mainly Ni_3_P and Cu_3_P phases. Compared with copper, nickel is more likely to form phosphides with P. After the reaction of nickel and phosphorus, the remaining part of the P has a chance to form new phosphides with copper.

The RXD data analysis results are consistent with the previous results. When the P content is low, only Ni_3_P phase appears, but, when the amount of P added exceeded the range in which the nickel can react, the remaining part of P form Cu_3_P phase with copper.

Figure 7 is the effect curve of adding different phosphorus content on the heat flow and sample temperature during the cooling process of ZCuPb20Sn5 alloy, (b) is the DTA curve of the area indicated of 1, and (c) is the area of 1 in the Fig. 7. It can be seen from the figure that the overall curve has three distinct exothermic peaks during the cooling process, and the first exothermic peak is between 935–975 °C, such as shown in Fig. 7(c), this peak should be the precipitation phase of the copper matrix, and the peak is relatively strong. The copper matrix precipitation freezing point is different with the P of different content. During the process from no P addition to 0.05% P addition, the solidification precipitation point of the copper matrix decreased from 961.77 °C to 945.05 °C, while the heat flow decreased from 0.93 μV to 0.037 μV. However, when the phosphorus content is increased to 0.1%, the peak point temperature suddenly increases to 974.67 °C, and then, as the phosphorus content increases, the temperature decreases.

The second exothermic peak is between 890–915 °C, as shown in Fig. 7(c). The melting point of (α + δ + Cu_3_P) eutectoid phase is 628 °C, copper is 1083 °C, and the formation temperature of δ phase is 520 °C, but the melting point of (Ni_3_P + Ni) eutectoid phase is 880 °C, So the second exothermic peak is the (α + δ + Ni_3_P) eutectoid precipitation point. When the amount of P added is less than 0.1%, the peak is mainly the precipitation point of the Cu-Sn solid solution.

The third exothermic peak is between 305 and 327 °C, as shown in Fig. 7(b). The melting point of lead is 328 °C, so this peak point is the point of the lead particle precipitation phase. The lead particle size is different with P different content added, so the temperature of lead particle precipitation is different. As the P content increases, the lead particle gradually becomes smaller, and thus the intensity of the peak gradually decreases. When phosphorus is added at 0.5 wt.%, the lead particles are the smallest, so the peak at this point basically disappears. For the precipitation temperature of this phase point, the P content of 0.1 wt.% is a turning point.The temperature decreases from 0% to 0.05%. Then, with the increase of P, the temperature point increases first and then decreases, the temperature is maximum at the point 0.2 wt.%.

**Figure 8:**
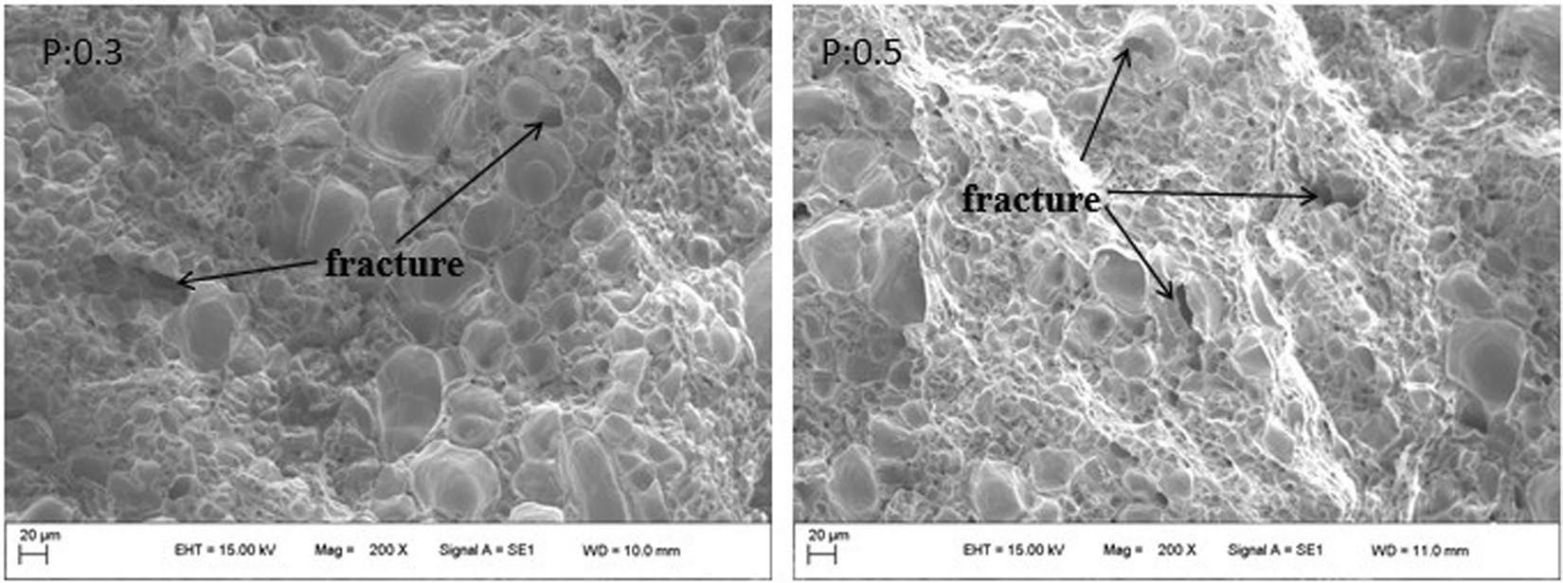
Tensile fractographs of ZCuPb20Sn5 alloy with different P.

Figure 8 shows a tensile fractograph of the P content of 0.3%wt.%t and 0.5%wt.%. It can be seen from the figure that both structures are ductile fractures. When adding P to 0.3%.wt, most of the middle part of the dimple is relatively large, and the middle part of the dimple becomes smaller with the P content of 0.5 wt.%. Only the individual large dimples are at the side, and the second phase particles are more, the dislocation ring is affected. The repulsion of the second phase particles, when subjected to an external force, the equilibrium is destroyed, and the dislocation loop is pushed toward the second phase particles, thus the interface begins to separate to form micropores. Generally, when the fracture conditions are the same, the dimple size is larger, indicating that the plasticity of the material is better. As can be seen from Fig. 8, the toughness of the alloy 0.5 wt.% P added is slightly worse than the 0.3%.

When the content of tin and P reached to a certain content, the Ni_3_P and (α + δ) phases formed the ternary eutectic phases (α + δ + Ni_3_P), which deteriorated the properties of the alloy to a certain extent. Therefore, the content of P in the modified tin-bronze alloy should not exceed 0.5 wt.%, otherwise it would cause hot cracking during processing.

## Conclusions

1. As the P content increased, the second phases Ni_3_P appeared and increased in size. The microstructure of ZCuPb20Sn5 alloy was refined and the elements were evenly distributed.
2. When the P content was less than 0.1 wt.%, the functions of phosphorous copper mainly was used as a deoxidizing initial gas, but when exceeded 0.1 wt.%, phosphorus formed a second phase reinforcing particle with copper or nickel together, which improved the mechanical properties of the alloy.
3. The second phase is mainly Ni_3_P, and a little Cu_3_P appeared only when the P content is greater than 0.3 wt.%. The strengthening effect of the second phase Ni_3_P improved the tensile strength and hardness of ZCuPb20Sn5.
4. The strength and hardness of ZCuPb20Sn5 alloy casting prepared by adding P 0.5 wt.% in melting process were 269.46 MPa and 108.18HB respectively, comparison with the strength and elongation of non-P refiner were 183.19 MPa and 64.2HB separately, they were increased by 47.1% and 68.5% respectively.When the amount of P was 0.5 wt.%, the tensile strength was 269.46 MPa. they were increased by 47.1% and 68.5% respectively. As the P content increased, the elongation rate increased first and consequently decreased, while the highest elongation was 18.65%.
